# AML Disparities Across Racial Ancestry Groups: A Spotlight on the NPM1 Mutations

**DOI:** 10.3390/ijms27010510

**Published:** 2026-01-03

**Authors:** Sarvath Aafreen Sanaullah, Pierre-Alexandre Vidi, Timothy S. Pardee

**Affiliations:** 1 Department of Cancer Biology, Wake Forest University School of Medicine, Winston Salem, NC 27157, USA; 2 Integrity of the Genome and Oncology (InGenO) Laboratory, Institut de Cancérologie de l’Ouest–ICO, 49055 Angers, France; 3 Department of Cancer Medicine, Wake Forest University School of Medicine, Winston Salem, NC 27157, USA

**Keywords:** acute myeloid leukemia, racial disparity, cancer health disparity, survival disparity

## Abstract

Racial and ethnic disparities in acute myeloid leukemia (AML) survival persist despite advances in treatment, with non-Hispanic black (NHB) patients and Hispanic patients often experiencing worse outcomes than Non-Hispanic White (NHW) patients due to a combination of clinical, socioeconomic, and biological factors. This review focuses on these disparities and emphasizes potential contributions of biology, as illustrated by the effects of the nucleophosmin 1 (NPM1) mutation. Mutation landscapes and chromosomal abnormalities strongly influence AML patient outcomes. While AML cases with NPM1 mutations are associated with favorable prognoses for NHW patients, NHB patients with NPM1-mutated AML have adverse outcomes. Thus, treatment algorithms and prognostic systems based on outcomes from a single racial ancestry group are inadequate. Beyond the more traditional socioeconomic determinants of health, addressing disparities in AML to achieve equity in care requires exploring biological factors linked to ancestry that shape treatment response.

## 1. Introduction

Acute myeloid leukemia (AML) is a heterogeneous disease characterized by a diverse set of genetic mutations and variable clinical outcomes [1]. The origin of AML involves the accumulation of mutations in hematopoietic stem or progenitor cells, leading to the uncontrolled proliferation of malignant myeloid cells. Risk stratification in AML is based on cytogenetic abnormalities and specific gene mutations [2]. Thus, next-generation sequencing and karyotyping is an essential component of AML diagnostic work up [2]. The prognostic value of these genetic changes is derived from clinical trials data examined retrospectively. The standard of care for AML often involves induction chemotherapy, with or without specific targeted treatments, with the goal of achieving a remission. Patients in remission then undergo consolidation therapy, including either repeated cycles of chemotherapy or allogeneic hematopoietic stem cell transplant (HSCT). Importantly, the decision to recommend HSCT in consolidation is based largely on the cytogenetic and molecular risk category of the patient. The incidence of AML varies across different racial groups, with higher incidence rates seen among White individuals compared to Black and Hispanic populations in the United States. Despite the survival advantage seen in certain genetic subtypes, Black and Hispanic patients experience significantly lower overall survival compared to White patients [3,4,5]. This review will examine the data on traditional factors of racial disparities, as well as possible biological differences by racial ancestry.

## 2. External Factors Contributing to AML Disparities

### 2.1. Clinical Trials Enrollment

Clinical trials are critical for the validation of new therapies for the treatment of cancer. They also provide data on which prognostic schemas (like the European Leukemia Net (ELN) 2022 risk classification system) are built, with the underlying assumption that outcomes in trial participants will be applicable to all AML patients. Currently, less than five percent of all adult cancer patients enroll in cancer clinical trials [6]. Clinical trial enrollment is impacted by several factors, such as limited awareness, insurance-related issues, distrust toward healthcare providers, especially in minority racial groups, socioeconomic barriers, and environment [7]. Several studies and meta-analyses of clinical trials in the past decade have shown that only 21 out of 90 AML clinical trials reported race or ethnicity of the participants. When race was reported, less than 5% were non-Hispanic black (NHB), and more than 80% of the enrolled participants were non-Hispanic white (NHW) [8,9,10,11,12,13,14]. Statler et al. (2019) investigated clinical trial enrollment at the Cleveland Clinic and found no difference in AML etiology or risk classification between NHB and NHW patients [15]. However, NHB patients were underrepresented in trials [15]. Similarly, another study noted that disparities in clinical trial enrollment, transplant access, and supportive care use contributed to racial differences in outcomes across hematologic malignancies [16]. Because treatment algorithms rely on clinical trial data, current AML care recommendations are largely shaped by outcomes from NHW patients. Worse survival outcomes are seen in minority racial groups compared to NHW patients with AML when treated with traditional cytotoxic regimens [17,18]. In contrast, emerging data suggest NHB patients may do better than NHW patients when treated with hypomethylating agents combined with venetoclax [19]. As a result of low minority clinical trial accrual, current treatment guidelines are biased towards optimizing the outcomes of NHW patients and do not consider the influence of racial ancestry on response and survival. 

### 2.2. Socioeconomic Factors

Structural inequities such as income, insurance status, and geographic access play a critical role in who receives the best care [20,21]. Retrospective cohort studies revealed that NHB patients experience significantly higher in-hospital mortality compared to their NHW counterparts, with disparities influenced by insurance status, hospital type, and patient comorbidities [22]. Byrne et al. (2011) showed that NHB patients, despite being younger at diagnosis, had significantly worse outcomes [23]. Key independent predictors of poor survival included Black race, Medicaid-only coverage, tobacco use, and residence in high-poverty areas [23]. These findings were corroborated by Abraham et al. (2018), who identified race and access to allogeneic transplants as independent predictors of survival in intermediate-risk AML [24]. Additionally, Bradley et al. (2022) found that, while Hispanic patients presented younger and had more adverse-risk cytogenetics (e.g., *KMT2A* rearrangements), when treated at academic institutions, their overall survival was not significantly different from NHW patients [25]. Hence, equal access to care may help mitigate some disparities. Despite improvements in survival rates, systemic inequities persist. Access to newer therapies such as venetoclax stay uneven, particularly in community settings, where older adults and individuals with higher socioeconomic status are more likely to receive advanced treatments [26]. These findings reflect ongoing challenges in fair access to therapeutic advancements for patients with lower socioeconomic status.

### 2.3. Environmental Factors

Additionally, environmental exposures and alterable lifestyle factors contribute significantly to the development and progression of AML. A study of 789 adult AML patients from six Chicago academic cancer centers found that NHB and Hispanic individuals experienced significantly higher exposure to air pollutants, including PAHPOM, PM2.5, diesel particulates, and benzene, compared to NHW patients [27]. PAHPOM exposure was independently associated with intermediate- and adverse-risk disease, suggesting a possible role of air pollution in disease biology [27]. Occupational chemical exposure, particularly to benzene, was associated with an increased risk of AML and myelodysplastic syndromes in a Minnesota-based population study [28]. Additionally, short-term exposures to vinyl chloride, creosote, and coal dust were positively correlated with AML risk [28]. In addition to occupational exposures, tobacco use can also contribute to AML risk. A meta-analysis including 7746 AML cases reported a 40% increased risk among current smokers and a dose-dependent relationship between smoking intensity, duration, and AML incidence [29]. A case–control study from Texas found significant associations between AML and solvent exposure, smoking, and obesity, with sex-specific and subtype-specific risk variations. The joint effect of solvent exposure and smoking further elevated risk [30]. Additionally, exposure to cytotoxic agents, electrical power sources, mental disorders, and family history of malignancy were identified as significant risk factors for AML [31]. These findings emphasize the importance of addressing environmental and occupational exposures, particularly in socioeconomically and racially marginalized populations, to reduce AML incidence and improve outcomes.

## 3. Mutations and Prognosis in AML

The molecular landscape of AML directly impacts its prognosis. The ELN has proposed cytogenetic risk stratification and response criteria. They currently describe three risk groups: favorable, intermediate, and adverse. As discussed above, these risk groups are based on large cohorts and clinical trial data that predominantly feature NHW patients. Likewise, studies validating risk stratification also use datasets that are dominated by NHW patients [32].

### 3.1. Favorable Risk Group

According to ELN 2022, approximately 30% of AML patients fall under the favorable risk category [33]. Studies show that patients in this risk group respond better to standard chemotherapy [2]. Genetic changes in this group include mutations in nucleophosmin-1 (NPM1) and the transcription factor CEBPA [34]. The five-year overall survival rate for these patients is over 50% [35]. Given this survival, HSCT is not recommended as consolidation therapy for these patients.

NPM1 mutations are among the most common in AML, present in roughly 30% of adult cases and up to 50–60% of cytogenetically normal AML (CN-AML) [36]. These mutations disrupt the nucleolar localization of the NPM1 protein, leading to its aberrant cytoplasmic accumulation, which contributes to leukemogenesis by dysregulating differentiation through the upregulation of homeobox genes (HOX). Cytoplasmic NPM1 blocks the migration of GADD45A to the nucleus, hindering its interaction with p53 to initiate apoptosis [37,38,39,40]. NPM1-mutated AML is categorized as favorable risk when FMS-like tyrosine kinase 3 internal tandem duplications (FLT3-ITD) is absent, according to the 2022 ELN classification [41,42]. However, relapses occur in up to 40% of NPM1-mutant (NPM1c) cases, suggesting the need for refined prognostic models incorporating measurable residual disease (MRD) and co-mutations [43]. Recent advances include the use of menin inhibitors, targeting the NPM1: HOX gene expression axis, which has shown preclinical and clinical promise for NPM1-mutant AML [44]. Revumenib, a menin inhibitor recently approved by the FDA for the treatment of AML with KMT2Ar, has also shown activity in NPM1-mutated AML patients and is now approved by the FDA for these patients as well [45]. As will be further discussed below, this improved prognosis appears to be specific to NHW patients. NHB patients with NPM1 mutations have outcomes that more closely resemble those of the adverse-risk category [3]. Despite these findings, the current standard practice is to not recommend HSCT for NHB patients with NPM1 mutations in first remission and highlights the need to incorporate patient specific outcomes, such as the presence of MRD at the time of remission, known to be highly prognostic in NMP1-mutated AML [46,47]. Guidelines emphasize the importance in obtaining MRD measurements. Therefore, MRD measurements in NPM1-mutated NHB patients are of great value.

CCAAT enhancer binding protein alpha (CEBPα) is a transcription factor regulating granulocytic differentiation. CEBPα has two transactivation domains in the N-terminal and one basic leucine zipper region (bZIP) in the C-terminal. The mutations are found in two hotspots, either insertion or deletions in the N-terminal or C-terminal. Frameshift in the N-terminal results in a dominant negative truncated protein, and the same mutation in the C-terminal is in-frame and results in the disruption of CEBPα to DNA or dimerization [48]. CEBPα mutations affect 5–10% of all AML cases [32,49]. Importantly, only mutations affecting the leucine zipper domain of the protein confer a favorable prognosis, and this is irrespective of whether this is mono- or bi-allelic [50]. Complete remission rates with chemotherapy are high in AML patients with these mutations. NHB patients have a lower frequency of CEBPA mutations [51].

Furthermore, translocations t(8;21) or inv(16)/t(16;16) are associated with the formation of novel fusion genes, leading to effects in the regulation of proliferation, differentiation, and viability of leukemic [52,53]. These translocations are seen in 5–10% of all AML cases and are associated with a favorable prognosis [52]. AML patients with these mutations are often treated with cytarabine-based intensive chemotherapy [53].

### 3.2. Intermediate-Risk Category

The ELN intermediate-risk category includes karyotypes and mutations not classified as favorable or adverse but does specifically include mutations in the receptor tyrosine kinase FLT3. As per ELN 2022 classification, 15–30% of patients fall into this category, and the five-year overall survival rate is less than 40% [35]. HSCT is recommended as consolidation for eligible patients who achieve a complete remission in induction therapy.

FLT3 mutations, particularly the internal tandem duplication (ITD), are associated with increased relapse and poor survival [54,55]. FLT3 mutations activate constitutive signaling pathways that promote proliferation and block apoptosis. They are often co-mutated with NPM1, worsening risk categorization and affecting approximately 10–20% of all adult AML cases [54]. FLT3 inhibitors have improved outcomes, especially in younger patients when combined with intensive chemotherapy [56]. The first-generation FLT3 inhibitor midostaurin was FDA approved in combination with intensive induction chemotherapy in 2017 [57]. The second-generation FLT3 inhibitor gliteritinib has clinical activity and is approved as a monotherapy for relapsed or refractory (R/R) AML [58,59]. Quizartinib, another second-generation FLT3 inhibitor, was more recently approved for combination therapy with intensive induction chemotherapy [60]. Resistance via secondary mutations and clonal evolution remains a challenge [61]. FLT3 mutations are also more prevalent in certain demographic groups. For instance, FLT3-ITD was detected more frequently in younger and female patients, though racial disparities in mutation prevalence remain inconclusive [62].

### 3.3. Adverse-Risk Category

AML patients under the adverse-risk category harbor autosomal monosomies involving chromosomes 5 or 7, complex karyotypes, mutations in certain tumor suppressors, epigenetic regulators, or spliceosome factors. As the name implies, adverse risk is associated with a worse overall survival rate (less than 10%) and usually affects older patients [35].

Around 2–8% of AML patients have TP53 mutations and are linked to complex karyotypes, therapy-related AML (t-AML), and poor outcomes. These mutations are common in elderly patients and those with prior exposure to chemotherapy or radiation [63]. TP53 mutations confer resistance to standard chemotherapy, hypomethylating agents, and venetoclax, making them a high-priority target for novel therapies [64]. While agents like eprenetapopt and magrolimab (anti-CD47) showed preliminary efficacy [64], confirmatory studies were disappointing. Eprenetapopt in combination with azacitidine failed to achieve the primary outcome in a phase 3 trial of TP53-mutant myelodysplastic syndrome (MDS), and magrolimab in combination with venetoclax and azacitidine was discontinued in phase 3 trials for both MDS and AML [65,66]. Although studies have been limited, a recent report found that NHB patients with TP53 mutations are younger and have more co-mutations than NHW patients [67]. Outcomes in TP53-mutated AML remain dismal regardless of racial ancestry, and there remains a large unmet medical need.

Other mutations contributing to the adverse-risk category include RNA splicing genes such as SRSF2, U2AF1, SF3B1, and ZRSR2, which causes aberrant splicing, resulting in AML, and is associated with unfavorable prognosis [68,69,70]. Furthermore, mutations in genes of the cohesin complex such as STAG1/2, SMC1/3, and RAD21 affect ~10% of AML patients and contribute to poor and adverse prognosis of the disease [70,71]. Frameshift mutations are mostly common in STAG2 and result in a loss of function causing abnormal differentiation of hematopoietic stem cells [71]. Chromatin modifiers such as EZH2, BCOR, and ASXL1 are mutated in AML and result in worse outcomes [70,72]. For currently unknown reasons, mutations in ASXL1 and BCOR are more common in NHB patients [51]. Currently, for adverse-risk AML, the only therapeutic modality with the ability to cure patients is HSCT, and this is recommended for all eligible patients.

## 4. Racial Ancestry and AML Biology

While many mutations are evenly distributed across races, trends suggest a higher prevalence of favorable-risk mutations (e.g.; NPM1) in NHW patients and lower rates in NHB and Hispanic populations [3,5] contributing to disparities in survival rates. Furthermore, minority populations continue to be underrepresented in clinical trials, limiting the generalizability of newer targeted therapies and risk models [73]. Despite the same treatment regimens, racial disparities in survival persist even among matched molecular risk patients, suggesting the influence of genetic ancestry [74]. Abraham et al. highlighted that Black patients experienced shorter survival even when treated under the same clinical trial protocols, suggesting that disease biologycontributes to the gap [24].

Several authors have explored how NPM1 mutation frequencies differ across racial groups (Table 1) [24,75]. Furthermore, Yunsuk et al. also noted that, while NPM1 mutations are prognostically favorable in general, their frequency and clinical impact may vary by race (summarized in Table 1) [75]. NPM1 mutations are not as frequently seen in NHB patients as in NHW patients [3,5]. Two independent studies by Bhatnagar et al. and Stiff et al. demonstrated that poor outcomes among NHB patients with AML are contradictorily associated with favorable-risk molecular profiles, including NPM1 mutations and wild-type FLT3 [3,5]. Given that NPM1 mutations are present in approximately one-third of AML cases, its clinical and prognostic significance is enormous. As discussed, treatment recommendations are influenced by risk classification, and currently, HSCT is recommended for intermediate and poor AML patients in first complete remission (CR1) [41,76]. This means that NPM1-mutated NHB patients treated with cytotoxic therapy who may benefit from HSCT in consolidation would not typically be recommended one under current guidelines. Additionally, NHB patients without high-risk mutations treated with a hypomethylating agent and venetoclax-based regimens showed improved survival compared to NHW patients [19]. Recently, the randomized PARADIGM study demonstrated that fit patients with intermediate- or adverse-risk AML had better outcomes when treated with azacitidine and venetoclax [77]. Given the classification of NPM1c as favorable risk, providers caring for NHB NPM1c patients may prioritize cytotoxic chemotherapy, as younger NPM1c patients were specifically excluded from this study. Prioritizing research investigating the molecular biology of NPM1c within different racial models to uncover ancestry-specific disease mechanisms is essential to narrow survival gaps and improve treatment recommendations. Survival rates of other minority racial groups also need to be further explored.

## 5. Molecular Properties of NPM1

NPM1 is a multifunctional protein localized in the nucleoplasm. It is a primary regulator of nucleolar architecture and function, coordinating ribosome biogenesis, stress responses, chromatin organization, and DNA repair. Each domain has a unique characteristic to allow interactions with molecules of important molecular pathways [78]. The protein’s dynamic localization, multivalent binding ability, and liquid–liquid phase separation (LLPS) highlight its involvement in multiple physiological and pathological processes.

### Structure of NPM1

**Figure 1:**
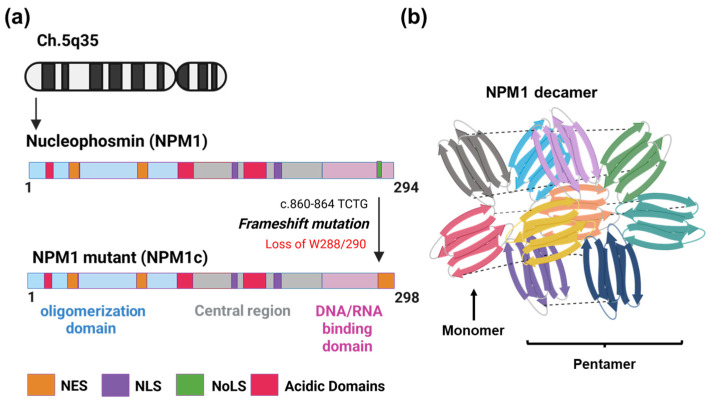
Structure and conformation of NPM1. (**a**) Gene and domain structure of NPM1 highlighting TCTG insertion, causing the frameshift mutation of the NPM1c mutant. NES, nuclear export signal; NLS, nuclear localization signal; NoLS, nucleolar localization signal. (**b**) Side view of NPM1 pentamers forming homo-decamers that facilitate molecular interactions.

The *NPM1* gene is found at ch5q35. The transcript has twelve exons. Varying splicing events result in two isoforms of the protein: NPM1.2 and NPM1.3 [78,79]. The primary transcript translates to a protein of 294 amino acids with a molecular weight of 37 kDa. NPM1 is comprised of three independent domains, an N-terminal hydrophobic domain for oligomerization to form homo-pentamers or heterodimers with a nuclear export signal (NES), a central domain with a nuclear localization signal (NLS), and a C-terminal domain allows for nuclear binding: both DNA and RNA and has a nucleolar localization signal (NoLS) (Figure 1a). NPM1 forms a homo-pentamer that is crucial for its activity [79]. X-ray crystallization of the NPM1 pentamer revealed that each monomer has two four-stranded anti-parallel β-sheets that are counter-aligned (Figure 1b). The β-strands connect in a sequential manner to form a pentamer. The far ends of the N- and C-termini in each monomer link together in a random manner for flexibility, and a loop between β2 and β3 of the core region joins to the first acidic tract. A decamer is formed when two pentamers align face to face and assemble into a closed ring, with each monomer from one pentamer connecting to a corresponding monomer from the other [78,79]. NPM1’s oligomeric feature facilitates its interaction with 130 proteins across various subcellular compartments. Through this feature, NPM1 forms pentamers with NPM1c and is transited to cytoplasm. Additionally, in the nucleolus, LLPS occurs through NPM1 condensates formation by its N-terminal oligomerization domain, hence further aiding in interactions with other proteins [80].

## 6. Role of NPM1 in Nucleolar Organization

NPM1 is a central organizer of the nucleolus through LLPS. It takes part in the formation of membrane-less compartments essential for ribosome biogenesis. Proteins such as SURF6 and ZNF692, as well as helicases like DDX24, modulate NPM1’s localization and dynamics within the granular part of the nucleolus. Alterations in these interactions affect the nucleolar structure and disrupt ribosome production [81,82]. One of the key regulators of NPM1 function is the long non-coding RNA LETN, recently identified as a primate-specific transcript that facilitates the assembly of NPM1 pentamers and nucleolar condensates. LETN’s scaffolding activity is essential for ribosomal RNA transcription and chromatin organization in both neural development and oncogenesis [83]. NPM1 holds intrinsically disordered regions (IDRs), particularly in the acidic domains and linker segments. These IDRs contribute to their ability to engage in multivalent and transient interactions with RNA, histones, and other proteins. Such interactions enable NPM1 to form a “fuzzy” molecular meshwork, essential for buffering proteotoxic stress and preventing aggregation of misfolded proteins [84]. Recent structural analyses have found specific aggregation-prone regions (APRs) within the NTD that predispose NPM1 to aggregation. These regions become particularly relevant in NPM1c+ mutants, which mislocalize to the cytoplasm in AML. This mislocalization is driven by the acquisition of a NES and loss of nucleolar retention motifs, leading to the formation of cytoplasmic aggregates that disrupt cellular homeostasis (also shown in Figure 1a) [40,85].

## 7. NPM1 in Genome Stability and DNA Repair

**Figure 2:**
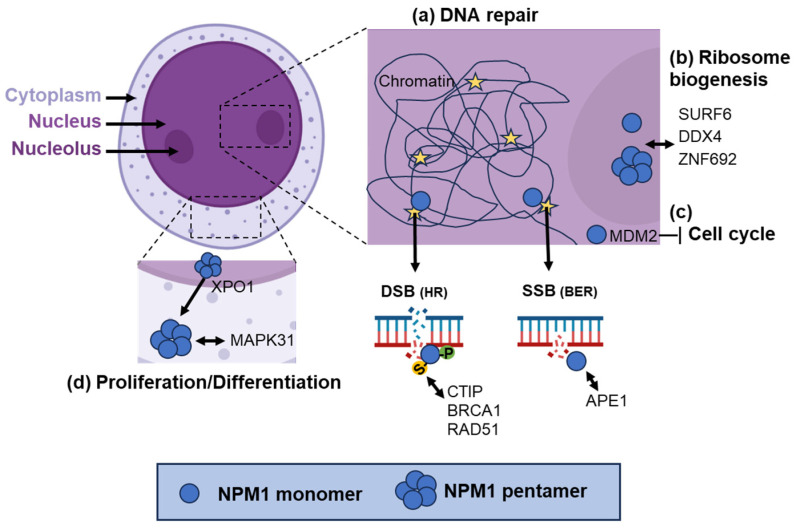
Molecular interactions of NPM1. (a) NPM1 is a DNA repair factor. Phosphorylation of NPM1 (-P) at T199 is mediated by CDK1/2 in the S phase and leads to NPM1 recruitment to DNA double-stranded breaks (DSBs), where the proteins participate in the initiation of repair by homologous recombination (HR): SUMOylation of NPM1 by Arf at K263 helps recruit HR factors, including CTIP, BRCA1, and RAD51. In single-stranded break (SSB) repair, NPM1 interacts with APE1 to facilitate the base excision repair (BER) pathway. (b) NPM1 is abundantly found in nucleoli and interacts with SURF6, DDX4, and ZNF692 for ribosome biogenesis and the formation of nuclear condensates. (c) NPM1 regulates MDM2 and is involved in the PIDDosome complex with caspase 2, resulting in cell cycle arrest and apoptosis. (d) NPM1 monomers and pentamers of wild-type (or mutated) NPM1 are exported to the cytoplasm via XPO1. There, NPM1 interacts with MAPK31 (part of the MAPKKK pathway), thereby impacting proliferation and differentiation.

NPM1 interacts with proteins involved in cell cycle regulation, chromatin remodeling, and tumor suppression to regulate protein stability, localization, and function through NoLS and multivalent binding domains. APE1 overexpression co-localizes with nucleolar NPM1 biomolecular condensates that recruit ATR, TopBP1, and ETAA1, thereby triggering cell cycle arrest and transcriptional silencing of rRNA genes [86,87]. In the nucleoplasm, NPM1 directly modulates the activity of enzymes involved in base excision repair (BER), such as APE1, Polβ, LigIII, and FEN1. It enhances APE1 endonuclease activity, ensuring the efficient repair of abasic sites (Figure 2) [87,88,89]. Phosphorylated NPM1 (pNPM1) plays a role in homologous recombination repair (HRR) (Figure 2) [90,91,92]. Cyclin-dependent kinases such as CDK1 and CDK2 phosphorylate NPM1 at T199 in the S phase [93,94]. NPM1 shuttles out of the nucleolus into the nucleoplasm and binds to DNA double-stranded break sites (DSBs) and colocalizes with γH2AX [95]. At the break sites, SUMOylation of NPM1 at K263 by SUMO1 helps in the recruitment of HRR proteins like CtIP, BRCA1, and RAD51 [96]. DeSUMOylation of NPM1 by SENP3 dislodges NPM1 from the break site for the DSB repair by HRR to occur successfully [97]. Furthermore, NPM1 stabilizes Polh (DNA polymerase eta), which is involved in trans-lesion synthesis (TLS), allowing replication to proceed past DNA lesions [87,98,99,100,101]. NPM1 also regulates the activity of DOT1L, a histone methyltransferase responsible for H3K79 methylation. Together, NPM1-DOT1L preserves chromatin architecture around the nucleoli and controls the expression of repetitive DNA sequences. Disruption of this axis results in heterochromatin mis-localization and compromised genome stability [102]. NPM1 participates in the PIDDosome complex, which senses centrosome amplification and triggers caspase-2 activation. This leads to MDM2 cleavage, p53 stabilization, and subsequent cell cycle arrest, ensuring genomic integrity in response to mitotic anomalies (Figure 2) [103]. Therefore, AML patients with aberrant cytoplasmic localization of NPM1 may have compromised nucleolar/nucleoplasm pathways, affecting their genomic integrity and promoting genomic abnormalites.

## 8. NPM1c in AML: Mutational Landscape and Functional Impacts

Mutations in NPM1, particularly frameshift mutations in exon 12, result in aberrant cytoplasmic localization (NPM1c), which is a defining feature in ~30% of AML cases. These mutations disrupt nucleolar retention and enable NPM1 to function abnormally in the cytoplasm and nucleoplasm, promoting leukemogenesis [85]. In NPM1-haploinsufficient mouse models, overexpression of MEIS1 is sufficient to induce AML, with MEIS1 aberrantly binding to the promoter of SMC4 and activating its transcription. This reprograms gene expressions in hematopoietic progenitors, contributing to transformation [104]. NPM1 mutations often cooccur with mutations in cohesin complex genes (e.g., SMC1A, RAD21, and STAG2). These mutations synergistically dysregulate HOXA/MEIS1 expression, promote enhancer hijacking, and block myeloid differentiation, establishing a transcriptional landscape conducive to leukemia [105,106,107]. After Etoposide treatment in the OCI-AML3 AML cell line (which has NPM1c), nucleolar re-localization of NPM1c is observed. This effect is specific to genotoxic drugs and has not been seen with agents acting via other mechanisms [108]. This indicates that NPM1c may retain the DNA repair roles mentioned above and may be partaking in nuclear/nucleoplasm mechanisms, raising awareness of a critical need for deeper investigation and targeting these pathways as a potential therapeutic strategy.

## 9. Therapeutic Implications and Strategies

NPM1’s centrality in AML pathogenesis makes it an attractive therapeutic target. NPM1c serves as a reliable biomarker for MRD monitoring and risk stratification [2]. Therapies targeting NPM1 aim to restore its nucleolar localization, disrupt its oligomerization, or harness the immune system against NPM1-derived neoantigens. NSC348884 is a small molecule that disrupts NPM1 oligomerization, leading to apoptosis in AML and non-small cell lung cancer (NSCLC) cells. This suggests a promising strategy to impair NPM1c+ functionality and enhance chemosensitivity [109]. Additionally, targeting LLPS has emerged as a novel strategy [110]. In adenovirus infection models, inhibiting NPM1-mediated LLPS hinders viral genome packaging [111,112]. Similarly, modulating interactions between NPM1 and binding partners like p14ARF or LETN may provide avenues to control proliferation in cancer or developmental conditions [83]. Menin-KMT2A inhibitors are a promising approach for targeting HOX-driven transcriptional programs in NPM1c AML [113]. These therapies may overcome resistance mechanisms and improve outcomes for patients with adverse-risk genetic profiles [114]. Finally, drugs that induce nucleolar stress, such as RNA polymerase I inhibitors, could exploit the vulnerability of cancer cells with hyperactive ribosome biogenesis [115,116]. These agents may synergize with therapies targeting NPM1 to enhance apoptotic responses. The use of Menin-KMT2A inhibitors or actinomycin D, especially in conjunction with MEIS1-SMC4 axis inhibitors, has shown synergy in suppressing AML growth in NPM1c contexts [86]. DNA-damaging agents like cytarabine induce the re-localization of NPM1c from the cytoplasm back to the nucleolus, restoring normal nucleolar functions and sensitizing AML cells to nucleolar stress [108]. How effective these strategies will prove to be in patients of differing racial ancestry is an important question.

## 10. Models to Investigate NPM1c in AML and Disease Progression

Currently, models for studying AML consist of immortalized cell lines, 3D models, and patient-derived xenografts (PDXs). At present, most AML models are of NHW ancestry, with only one cell line of African descent, and it does not contain NPM1c [117,118]. To investigate the role of NPM1c in AML and disease progression, there are no available models of African ancestry. Although primary patient sample cell cultures have been used to study AML, the genetic and functional heterogeneity among different AML samples make the results inconsistent and unreliable [119,120]. In AML, only 25% of all NHB AML patients harbor NPM1 mutations; thus, acquiring samples for primary cell culture and establishing cell lines is challenging [3]. Therefore, increased efforts must be made to generate pre-clinical models of NPM1c using patient-derived xenografts or NPM1c-induced AML in hematopoietic stem cells (HSCs) of NHB racial ancestry. These models are needed to address the influence of NHB racial ancestry on the mechanisms and pathways of NPM1c-mediated leukemogenesis and response to therapy.

## 11. Conclusions

Disparities in AML survival across racial and ethnic groups are rooted in clinical, social, and biological factors. While socioeconomic barriers and unequal access to care are known, emerging evidence highlights that disease biology may differ by ancestry. Closing the survival gap in AML will require changes across biomedical research, clinical practice, and public health to create a fairer healthcare system. Considering that AML with NPM1 mutations is associated with favorable prognosis when treated with DNA damaging chemotherapy in NHW patients but not NHB, we should prioritize research on NPM1’s substantial role in DNA repair, particularly through the lens of ancestral background. NPM1, as a driver of AML, is a promising target for therapeutic strategies focusing on inhibiting oligomerization, disrupting its protein–protein interactions, and regulating its role in phase-separated nuclear compartments. The lack of appropriate preclinical models has hindered the investigation of DNA repair mechanisms in AML with mutated NPM1. Future clinical trials must include participants across different ancestral groups with pre-planned subgroup analysis to ensure ancestry-specific responses can be determined when present. The current approach of applying results from a predominantly NHW population and assuming similar efficacy in all groups must be replaced. This will pave the way for the development of next-generation targeted therapies that are both precise and inclusive, improving outcomes for underrepresented populations.

## Figures and Tables

**Table 1:** Frequency of NPM1c mutations in different ethnicities.

	Non-Hispanic Whites NPM1c Frequency	Non-Hispanic Blacks NPM1c Frequency	Hispanic NPM1c Frequency	Non-Hispanic NPM1c Frequency	Outcomes	Therapy
Bhatnagar et al. [3]	38% (*n* = 777)	25% (*n* = 72)	-	-	Worse OS in NHB patients	Intensive Chemotherapy
Andrew et al. [5]	29.3% (*n* = 323)	20% (*n* = 100)	-	-	Worse OS in NHB patients	Intensive Chemotherapy
Mahmood et al. [73]	-	-	6.9% (*n* = 58)	10.8% (*n* = 167)	No differences in survival	-
Abraham I et al. [24]	47% (*n* = 59)	31% (*n* = 18)	25% (*n* = 17)	-	Better OS in NHW patients	Intensive or HMA treatment
Wang et al. [19]	10% (*n* = 870)	6% (*n* = 61)	13% (*n* = 45)	-	Better OS in NHB patients	Venetoclax based low intensity induction therapy

## Data Availability

No new data were created or analyzed in this study. Data sharing is not applicable to this article.

## Abbreviations

The following abbreviations are used in this manuscript:

NHB	Non-Hispanic Black
NHW	Non-Hispanic White
NPM1	Nucleophosmin 1
AML	Acute myeloid leukemia
HSCT	Allogeneic hematopoietic stem cell transplant
ELN	European Leukemia Net
CN-AML	Cytogenetically normal AML
HOX	Homeobox genes
MRD	Measurable residual disease
CEBPα	CCAAT enhancer binding protein alpha
bZIP	Basic leucine zipper region
FLT3-ITD	FMS-like tyrosine kinase 3 internal tandem duplication
t-AML	Therapy-related AML
MDS	Myelodysplastic syndrome
CR1	Complete remission
LLPS	Liquid–liquid phase separation
NES	Nuclear export signal
NLS	Nuclear localization signal
NoLS	Nucleolar localization signal
IDRs	Intrinsically disordered regions
APRs	Aggregation-prone regions
BER	Base excision repair
pNPM1	Phosphorylated NPM1
HRR	Homologous recombination repair
DSBs	DNA double-stranded break sites
TLS	Trans-lesion synthesis
NSCLC	Non-small cell lung cancer
NPM1c	Cytoplasmic NPM1
PDXs	Patient-derived xenografts
HSCs	Hematopoietic stem cells
